# Skin picking disorder and hazardous alcohol use

**DOI:** 10.1017/S1092852925100813

**Published:** 2025-12-12

**Authors:** Jon E. Grant, Sophie Boutouis

**Affiliations:** 1 The University of Chicago, USA; 2 Psychiatry and Behavioral Neuroscience, The University of Chicago Medicine, USA

**Keywords:** Skin picking, excoriation, alcohol, impulsivity, aggression

## Abstract

**Background:**

Skin picking disorder is characterized by repetitive excoriation of one’s skin. Although skin picking disorder is associated with substance use problems, no previous research has examined the associations of alcohol use on skin picking symptomatology.

**Methods:**

Adults with skin picking disorder (*n* = 182) were recruited from the general community via an online survey. Participants completed the Alcohol Use Disorders Identification Test (AUDIT) to measure alcohol use and various self-report measures to assess clinical profiles and associated characteristics. We compared variables of interest between those with hazardous alcohol use in the past year (AUDIT ≥8) compared to those with low-risk or no alcohol use in the past year. We also examined the extent to which skin picking disorder symptoms were dimensionally related to AUDIT scores while controlling for confounders.

**Results:**

Of the 182 adults with skin picking disorder, 62 (34.1%) met criteria for hazardous alcohol use. Hazardous drinking was associated with less frequent skin picking but higher overall picking-related severity and impairment, aggression, and rates of comorbid borderline personality disorder, gambling disorder, and compulsive sexual behavior disorder. Skin picking-related impairment predicted the degree of hazardous drinking while controlling for sex.

**Conclusions:**

This study highlights the importance of screening for hazardous alcohol use in people with skin picking disorder. More research is needed to explore the relationship between aggression, hazardous alcohol use, and skin picking, as well as how treatments might best be adapted to treat individuals with this cluster of symptoms.

## Highlights

- Hazardous alcohol use was associated with less frequent skin picking but more overall impairment and severity.
- Hazardous alcohol use was associated with greater aggression.
- Those with hazardous alcohol use were more likely to meet criteria for a range of impulsive disorders.

## Introduction

Skin picking disorder is characterized by the failure to resist impulses to pick one’s skin, often resulting in excoriations, scarring, and even infections (American Psychiatric^1^). People with skin picking disorder have high rates of current and lifetime psychiatric comorbidities and tend to report problems with impulsive behaviors.^2^ Research has suggested that skin picking disorder may be associated with externalizing behaviors such as alcohol use.^3^ Further support for this association between alcohol use and skin picking comes from studies examining rates of co-occurring hazardous alcohol use in adults with skin picking disorder (31.7% in^4^; 25.0% in^5^; 16.0% in^6^). These studies report rates of current hazardous alcohol use, which are higher than those reported in the U.S. population (ie, 13.2% of adults).^7^

People with skin picking disorder often experience strong urges to pick their skin, heightened distress, and increased rates of psychiatric disorders associated with poor distress tolerance and aggression (one form of which is harming oneself).^2^^,^^8^ In terms of aggression, there is a robust literature examining the complex relationship between alcohol use and aggression (ie, aggression seen in intoxication,^9^ withdrawal,^10^ and even abstinence^11^). Therefore, it is possible that in some people with skin picking disorder, aggression and alcohol use may be associated with their skin picking behavior. To our knowledge, however, no research to date has examined the relationship between hazardous alcohol use, aggression, and skin picking symptoms.

In the current study, we use data collected from an online survey to (1) quantify rates of past-year hazardous alcohol use in adults with skin picking disorder in a sample from a community sample; (2) examine whether the degree of alcohol use is related to skin picking disorder severity and impairment; and (3) assess rates of aggression and comorbidities in those with and without hazardous alcohol use in the sample. We hypothesized that those with hazardous alcohol use would report greater skin picking disorder severity and impairment, and greater aggression, relative to those with no alcohol problems.

## Methods

### Participants

Participants included 182 adults who self-identified as having skin picking disorder and were recruited from the community through Reddit support groups for obsessive–compulsive disorder, body-focused repetitive behaviors (BFRBs), and skin picking disorder. The survey was advertised by posting in these groups with a link to the survey, and it was not circulated to other groups. We adopted this recruitment approach because people with skin picking disorder appear to be highly active in Reddit support groups for their condition. Inclusion criteria for the clinical sample were (a) a DSM-5 diagnosis of skin picking disorder; (b) aged 18–65 years; (c) fluent in English; and (d) capable of providing informed consent. Participants were excluded if they were unable to understand or provide informed consent.

### Procedure

The Institutional Review Board of the University of Chicago approved the study and the consent statement (IRB #21-1267). All procedures contributing to this work comply with the ethical standards of the relevant national and institutional committees on human experimentation and with the Declaration of Helsinki 1975, as revised in 2008.

First, participants read the online informed consent page and chose to participate in the survey or opt out. This page told all participants that the information they provided was confidential. Participants were compensated at the end of the survey using random prize drawings. Those completing the survey were entered into a prize drawing, and 15 participants were randomly chosen to receive a $100 gift certificate. Participants’ contact information for the prize drawings was stored separately from their survey responses to protect their privacy. Three hundred one individuals with skin picking disorder completed the survey. Of the respondents, 14 did not report their alcohol use, and 105 had comorbid trichotillomania (which could have interfered with our measure of “global” BFRB severity), so they were excluded from the analyses. As such, the final sample consisted of 182 adults. The survey was conducted via REDCap. Quality checks were performed through rule logic, and REDCap automatically excluded users who had already completed the survey and calculated the time taken for survey completion (participants completing the survey in < 10 min were flagged and excluded, as < 10 min for completion was unrealistic). Duplicate or highly similar responses were reviewed using the data comparison module on REDCap. All responses were also checked individually for inconsistencies. The survey was open from April 20, 2023 to May 11, 2023.

### Assessments

Participants were assessed for age, sex, race, education, and sexual orientation. Each participant completed the previously validated self-report version of the Minnesota Impulse Disorders Interview version 2.0 (MIDI 2.0)^12^^,^^13^ to verify the diagnosis of skin picking disorder (using questions that mirror the DSM-5 criteria). They were also asked to report the number of days per week and time per day they spent picking their skin. Participants completed the *Alcohol Use Disorders Identification Test (AUDIT), a validated screening tool for hazardous alcohol use* (scores of ≥8 indicate hazardous alcohol use) to examine past-year alcohol use^14^ (α = 0.945 in the present study). Possible scores range from 0 to 40, with higher scores suggesting more hazardous drinking.

To measure skin picking disorder symptoms, participants completed the Generic Body-Focused Repetitive Behavior Scale-8 (GBS-8), an 8-item self-report scale measuring the severity and impairment of BFRBs (α = 0.808 in the current study).^15^ Total scores on the GBS-8 range from 0 to 32 (higher scores indicate worse outcomes), and symptom-severity and impairment-subscale scores were also calculated. Since the GBS-8 can evaluate multiple BFRBs, we restricted our analyses to those with skin picking disorder only. Survey respondents who endorsed comorbid trichotillomania were excluded.

Participants also completed the Brief Aggression Questionnaire (BAQ), a 12-item self-report measure of trait aggression (α = 0.868 in the present study). The BAQ measures aggression on the dimensions of physical aggression, verbal aggression, anger, and hostility,^16^ as aggression has been linked to alcohol use, impulsivity, and obsessive–compulsive spectrum disorders.^17^ Higher subscale scores and total scores indicate more aggression.

In terms of psychiatric comorbidities, participants were screened for probable borderline personality disorder (BPD), post-traumatic stress disorder (PTSD), and impulse-control disorders. First, they completed the BPD module of the Personality Assessment Inventory, which screens for core features of BPD^18^ (α = 0.850 in the current study). This scale includes 24 items rated on a 4-point Likert scale (0 = false to 3 = very true), with total scores of ≥38 indicating significant features of BPD. Then, participants underwent the Primary Care PTSD Screen for DSM-5 (PC-PTSD-5) (a screen for PTSD; total scores ≥5 indicate likely PTSD) (α = 0.908 in the present study).^19^ To better understand the role of impulsive conditions in skin picking disorder, each participant completed the MIDI 2.0.^12^^,^^13^ This interview screens for compulsive buying disorder, kleptomania, gambling disorder, binge-eating disorder, and compulsive sexual behavior disorder.

### Statistics

The percentage of participants who met criteria for past-year hazardous alcohol use (score of ≥8 on the AUDIT) was determined. These participants were compared to those without past-year hazardous drinking on measures of clinical severity, aggression, and comorbidities. Between-group differences were tested using either independent samples *t* tests, analyses of covariance (ANCOVAs), or Pearson’s χ^2^ or Fisher’s exact tests. Statistical significance was set at *p* < .01 for these tests to correct for multiple comparisons. Effect sizes were also calculated. Effect sizes for the equality of mean differences between groups were reported in terms of Cohen effect size index (“d”) for *t* tests, where a d of 0.2 is conventionally considered a small effect size, 0.5 is medium, and .8 is large. For ANCOVAs, partial eta squared was reported (0.01 = small effect size, 0.06 = medium effect size, 0.14 = large effect size), and for chi-square tests, the Phi coefficient (0.1 = weak, 0.3 = moderate, 0.5 = strong) was calculated to measure effect size. A multiple linear regression assessed whether skin picking disorder symptoms were dimensionally related to the degree of hazardous drinking after controlling for confounders.

## Results

The study comprised 182 adults with skin picking disorder (mean age = 29.92, *SD* = 8.82 years, range = 18–64 years; 80.8% female). The demographics for the entire sample are presented in Table 1.

**Table 1.** Descriptive Statistics for a Sample of 182 Adults with Skin Picking Disorder

		*M (SD)* or %
Age		29.92 (8.82)
Sex	Female	80.8
	Male	18.1
	Intersex	1.1
Race	White	94.0
	Non-White	6.0
Education	High school or less	7.7
	Some college	24.2
	Trade school	11.5
	College	32.4
	Grad school	24.2
Sexual orientation	Heterosexual	62.1
	Gay or lesbian	6.6
	Bisexual	22.5
	Other	8.8
Days spent picking skin per week	3–4 days	26.9
	5–6 days	11.0
	7 days	62.1
Time spent picking skin per day	16–30 minutes	27.5
	31–60 minutes	30.2
	1–2 hours	26.9
	2+ hours	15.4

Of the 182 adults with skin picking disorder, 62 (34.1%) scored ≥8 on the AUDIT, indicating hazardous alcohol use. Those skin picking disorder adults who were hazardous drinkers did not significantly differ from nonhazardous drinkers with respect to age or race (both *p* > 0.01). Those with hazardous alcohol use were, however, more likely to be male (χ^2^ = 30.543, *p* < 0.001), have less formal education (χ^2^ = 16.328, *p* = 0.003), and identify as heterosexual (χ^2^ = 14.610, *p* = 0.002) (Table 2).

**Table 2.** Descriptive Statistics for a Sample of 182 Adults With Skin Picking Disorder, Stratified by Alcohol Use

		Group		*t* or χ^2^	*p*	Cohen’s d or Phi
		Nonhazardous alcohol use (*n* = 120), *M (SD)* or %	Hazardous alcohol use (*n* = 62), *M (SD)* or %			
Age		29.43 (9.29)	30.85 (7.82)	−1.089	0.278	−0.161
Sex	Female	93.2	59.7	30.543	<0.001	0.412
	Male	6.8	40.3			
Race	White	94.2	93.5	0.028	0.868	−0.012
	Non-White	5.8	6.5			
Education	High school or less	9.2	4.8	16.328	0.003	0.300
	Some college	20.8	30.6			
	Trade school	6.7	21.0			
	College	32.5	32.3			
	Grad school	30.8	11.3			
Sexual orientation	Heterosexual	52.5	80.6	14.610	0.002	0.283
	Gay or lesbian	9.2	1.6			
	Bisexual	28.3	11.3			
	Other	10.0	6.5			

Bolded characteristics are statistically significant predictors of the outcome variable (*p* < 0.01).

When compared to the participants with skin picking disorder without hazardous alcohol use, those with hazardous alcohol use reported significantly fewer days per week of picking their skin (χ^2^ = 64.282, *p* < 0.001) after controlling for sex. However, the groups did not differ in the time they spent picking their skin per day (Table 3). Additionally, those with hazardous alcohol use had greater overall skin picking severity based on the GBS-8 total score (*M* = 19.81, *SD* = 4.90 compared to *M* = 16.55, *SD* = 3.67, *F*(1, 177) = 21.486, *p* < 0.001), and greater impairment due to their skin picking (*M* = 9.60, *SD* = 2.61 vs. *M* = 6.81, *SD* = 2.35, *F*(1, 177) = 45.477, *p* < 0.001). Additionally, those with hazardous alcohol use had higher scores of total aggression (*M* = 4.02, *SD* = 0.950 compared to *M* = 2.94, *SD* = 1.02, *F*(1, 170) = 32.304, *p* < 0.001) and higher scores on all subscales of aggression except hostility (Table 3).

**Table 3.** Measures of Skin Picking, Aggression, and Comorbidities for a Sample of 182 Adults With Skin Picking Disorder, Stratified by Alcohol Use

	Control variable		Group		*F* or χ^2^	*p*	η^2^ or φ
			Nonhazardous alcohol use (*n* = 120), *M (SD)* or %	Hazardous alcohol use (*n* = 62), *M (SD)* or %			
GBS–8 severity score			9.75 (2.13)	10.21 (2.56)	1.397	0.239	0.008
GBS–8 impairment score			6.81 (2.35)	9.60 (2.61)	45.477	<0.001	0.204
GBS–8 total score			16.55 (3.67)	19.81 (4.90)	21.486	<0.001	0.108
Days spent picking skin per week	**Females**	3–4	5.5	45.9	34.967	<0.001	0.488
		5–6	11.8	10.8			
		7	82.7	43.2			
	Males	3–4	37.5	88.0	8.643	0.013	0.512
		5–6	12.5	4.0			
		7	50.0	8.0			
	**Total**	3–4	7.6	62.9	64.282	<0.001	0.598
		5–6	11.9	8.1			
		7	80.5	29.0			
Time spent picking skin per day	Females	16–30 minutes	27.3	29.7	1.798	0.615	0.111
		31–60 minutes	32.7	21.6			
		1–2 hours	22.7	29.7			
		2+ hours	17.3	18.9			
	Males	16–30 minutes	12.5	24.0	3.907	0.272	0.344
		31–60 minutes	12.5	40.0			
		1–2 hours	62.5	32.0			
		2+ hours	12.5	4.0			
	Total	16–30 minutes	26.3	27.4	0.927	0.819	0.072
		31–60 minutes	31.4	29.0			
		1–2 hours	25.4	30.6			
		2+ hours	16.9	12.9			
AUDIT total score			1.92 (1.81)	18.68 (7.46)	435.156	<0.001	0.711
BAQ physical aggression score			2.27 (1.33)	3.87 (1.44)	37.451	<0.001	0.180
BAQ anger score			2.63 (1.39)	3.85 (1.11)	24.413	<0.001	0.124
BAQ verbal aggression score			3.25 (1.36)	4.07 (1.24)	9.253	0.003	0.051
BAQ hostility score			3.68 (1.46)	4.29 (1.16)	6.674	0.011	0.037
BAQ total score			2.94 (1.02)	4.02 (.950)	32.304	<0.001	0.160
Borderline personality disorder	Females	No	76.1	75.0		1.00	0.009
		Yes	23.9	25.0			
	**Males**	No	80.0	0.0		0.004	0.853
		Yes	20.0	100.0			
	**Total**	No	76.3	40.9		0.003	0.318
		Yes	23.7	59.1			
Post-traumatic stress disorder	Females	No	91.8	85.7		0.327	0.088
		Yes	8.2	14.3			
	Males	No	87.5	75.0		0.646	0.131
		Yes	12.5	25.0			
	Total	No	91.5	81.4		0.082	0.148
		Yes	8.5	18.6			
Buying disorder	Females	No	88.6	73.0		0.034	0.189
		Yes	11.4	27.0			
	Males	No	87.5	87.5		1.00	0.000
		Yes	12.5	12.5			
	Total	No	88.5	78.7		0.117	0.131
		Yes	11.5	21.3			
Kleptomania	Females	No	100	97.3		0.261	0.142
		Yes	0	2.7			
	Males	No	87.5	95.8		0.444	−0.149
		Yes	12.5	4.2			
	Total	No	99.1	96.7		0.281	0.088
		Yes	0.9	3.3			
Gambling disorder	**Females**	No	100	78.4		<0.001	0.412
		Yes	0	21.6			
	Males	No	87.5	83.3		1.00	0.050
		Yes	12.5	16.7			
	**Total**	No	99.1	80.3		<0.001	0.341
		Yes	0.9	19.7			
Binge-eating disorder	Females	No	87.6	86.5		0.1.00	0.015
		Yes	12.4	13.5			
	Males	No	75.0	95.8		0.147	−0.309
		Yes	25.0	4.2			
	Total	No	86.7	90.2		0.629	−0.050
		Yes	13.3	9.8			
Compulsive sexual behavior disorder	**Females**	No	97.1	75.7		<0.001	0.339
		Yes	2.9	24.3			
	Males	No	87.5	58.3		0.209	0.266
		Yes	12.5	41.7			
	**Total**	No	96.5	68.9		<0.001	0.389
		Yes	3.5	31.3			

Bolded characteristics are statistically significant predictors of the outcome variable (*p* < 0.01). Sex was controlled for in all analyses.

Abbreviation: AUDIT, Alcohol Use Disorders Identification Test; BAQ, Brief Aggression Questionnaire; GBS-8, Generic Body-Focused Repetitive Behaviors Scale-8 items.

Those participants with skin picking disorder who were categorized as hazardous drinkers significantly differed from those without hazardous drinking with respect to greater rates of BPD (59.1% vs. 23.7%, *p* = 0.003), gambling disorder (19.7% compared to 0.9%, *p* < 0.001), and compulsive sexual behavior disorder (31.1% vs. 3.5%, *p* < 0.001). After controlling for sex, we determined that the elevated rates of BPD were driven by males (*p* = 0.003), and the increased rates of gambling and compulsive sexual behavior disorder were driven by females (both *p* < 0.001). Rates of buying disorder, kleptomania, and binge-eating disorder did not significantly differ between groups (Table 3).

GBS-8 severity (*r* = 0.150, *p* = 0.043) and impairment (*r* = 0.512, *p* < 0.001) scores were positively correlated with AUDIT scores. After controlling for sex (0 = female, 1 = male) in a multiple linear regression, GBS-8 impairment scores were still associated with hazardous drinking in the form of AUDIT scores (*t* = 7.444, *p* < 0.001). However, GBS-8 severity scores were not significant predictors of AUDIT scores (Table 4). The overall utility of the model was significant, *F*(3,176) = 33.153, *p* < 0.001, *R^2^* = 0.350.

**Table 4.** Multiple Linear Regression Evaluating Predictors of AUDIT Total Scores

Predictors				
	Standardized coefficients β	*t*	95% CI	*p*
Sex	.287	4.671	3.932–9.684	<0.001
GBS–8 severity score	−.132	−1.886	−1.086 to .025	0.061
GBS–8 impairment score	.530	7.444	1.293–2.226	<0.001

Bolded characteristics are statistically significant predictors of the outcome variable (*p* < 0.01).

Abbreviation: AUDIT, Alcohol Use Disorders Identification Test; GBS-8, Generic Body-Focused Repetitive Behaviors Scale-8 items.

## Discussion

This study is the first that we are aware of that examined hazardous alcohol use and its clinical associations in adults with skin picking disorder. The rate of past-year hazardous alcohol use in adults in this study (34.1%) is notably higher than reported in previous studies of skin picking disorder^4^^,^^6^ as well as higher than the rate found in the general U.S. population (ie ,13.2% of adults).^7^ Interestingly, the rate here is also much higher than that reported in trichotillomania (13.2%)^20^ and highlights a potentially important difference between skin picking disorder and hair pulling.

Regardless of the specific causal relationship between skin picking disorder and hazardous alcohol use, the fact that they frequently co-occur raises important clinical issues. For one, the rate of hazardous drinking in skin picking disorder suggests that clinicians should carefully screen people with skin picking disorder for hazardous alcohol use, as treatment of skin picking disorder could be compromised by the presence of untreated hazardous alcohol use. It is also worth noting that the pattern of skin picking appears to be less frequent but more severe in people with hazardous drinking. Thus, clinicians screening for this relationship should be aware of this pattern, as it may often go unnoticed if both the picking and the alcohol use are episodic. We are unaware of any research that has examined the treatment of comorbid hazardous alcohol use in patients with skin picking disorder, but the question of whether a pharmacological intervention, such as naltrexone, might be useful for both simultaneously might be worth investigating.^21^

The new finding of this study was that aggression was significantly more pronounced in those participants with skin picking disorder and hazardous alcohol use. There are potentially multiple explanations for these associations. Without data regarding the temporal relationship between alcohol intoxication/withdrawal, aggression, and the picking behavior, (a limitation of this study but potentially an important future research focus), it is possible that a variety of variables could drive these associations. For example, poor distress tolerance developmentally may lead to picking, aggression, and alcohol use as means of coping. Conversely, hazardous alcohol use may in turn lead to both heightened aggression and picking (as picking may be a form of self-aggression in some individuals). This study is the first to show that there is a correlation between worsening hazardous alcohol use and skin picking severity. It also lays a foundation for examining other important variables (eg, aggression, distress intolerance as reflected in BPD) to better understand this relationship. It is also possible that aggression may aggression may drive a unique profile of skin picking disorder that is characterized by more extreme (ie aggressive) but less frequent skin picking behaviors. Future research therefore may want to understand the temporal relationship of these variables to more accurately treat, if possible, the driving force of the behaviors and to craft public health initiatives to thwart the development of the secondary problems.

This study has several limitations. Most notably, we based hazardous alcohol use on subject report only. Because substance use is often denied, the rates found in this study may actually underestimate the actual rate of hazardous alcohol use in patients with skin picking disorder. Conversely, the AUDIT is a screen, and as such, may over-pathologize behaviors; therefore, these rates may be somewhat higher than would warrant psychosocial interventions. Given the survey nature of this study, clearly no one-on-one diagnostic interviews were conducted to verify hazardous alcohol use. On a related note, we did not collect age of onset for alcohol use and this may confound our results. Early onset drinking can actually disrupt neurodevelopmental processes to generate a more impulsive profile and thus could be a causal factor in both skin picking disorder and hazardous drinking. If this were the case, targeting early onset drinking would be the key target, and future studies should therefore take into account all the key confounders. Second, we used screens for probable diagnoses of BPD and PTSD and thus these measures report only BPD traits or possible diagnosis of PTSD. As the assessment was conducted with an online survey, these probable diagnoses are self-reported. Third, we excluded people with comorbid trichotillomania. Trichotillomania has a comorbidity rate of approximately 20–25% in people with skin picking disorder,^22^ and so our resulting sample might be argued to not be representative of skin picking disorder generally. Fourth, there were no temporal data regarding use of alcohol and picking episodes and the onset of the co-occurring disorders. Future research that investigates the temporal relationship between alcohol use, skin picking disorder, and comorbid disorders seems crucial for our understanding of the impact of alcohol on skin picking and comorbid disorders. Finally, participants in this study were a nonclinical population, predominantly Caucasian and female, which may lessen the generalizability of this study to participants of other races and genders. Furthermore, as this is a survey study and we cannot verify the information regarding their behaviors, or the diagnosis of skin picking disorder beyond the MIDI screen, this study sample may not be representative of all individuals with skin picking disorder.

In conclusion, rates of hazardous alcohol use are elevated in skin picking disorder compared with the general population and therefore merit further evaluation and understanding. The picture that appears to be coming into view is that a large percentage of people with skin picking disorder may struggle with unhealthy levels of alcohol use and aggression. Examining the temporality of these variables to target the underlying issues may be worthy of further investigation.
